# Comparison of a Short Linear Antimicrobial Peptide with Its Disulfide-Cyclized and Cyclotide-Grafted Variants against Clinically Relevant Pathogens

**DOI:** 10.3390/microorganisms9061249

**Published:** 2021-06-08

**Authors:** Johannes Koehbach, Jurnorain Gani, Kai Hilpert, David J Craik

**Affiliations:** 1 Australian Research Council Centre of Excellence for Innovations in Peptide and Protein Science, Institute for Molecular Bioscience, The University of Queensland, Brisbane, QLD 4072, Australia; j.koehbach@uq.edu.au (J.K.); d.craik@imb.uq.edu.au (D.J.C.); 2 Institute for Infection and Immunity, St. George’s University, London SW17 0RE, UK; jgani@sgul.ac.uk

**Keywords:** antimicrobial peptide, cyclotide, cysteine cyclization, ESKAPE, serum activity, stability, MCoTI-II

## Abstract

According to the World Health Organization (WHO) the development of resistance against antibiotics by microbes is one of the most pressing health concerns. The situation will intensify since only a few pharmacological companies are currently developing novel antimicrobial compounds. Discovery and development of novel antimicrobial compounds with new modes of action are urgently needed. Antimicrobial peptides (AMPs) are known to be able to kill multidrug-resistant bacteria and, therefore, of interest to be developed into antimicrobial drugs. Proteolytic stability and toxicities of these peptides are challenges to overcome, and one strategy frequently used to address stability is cyclization. Here we introduced a disulfide-bond to cyclize a potent and nontoxic 9mer peptide and, in addition, as a proof-of-concept study, grafted this peptide into loop 6 of the cyclotide MCoTI-II. This is the first time an antimicrobial peptide has been successfully grafted onto the cyclotide scaffold. The disulfide-cyclized and grafted cyclotide showed moderate activity in broth and strong activity in 1/5 broth against clinically relevant resistant pathogens. The linear peptide showed superior activity in both conditions. The half-life time in 100% human serum was determined, for the linear peptide, to be 13 min, for the simple disulfide-cyclized peptide, 9 min, and, for the grafted cyclotide 7 h 15 min. The addition of 10% human serum led to a loss of antimicrobial activity for the different organisms, ranging from 1 to >8-fold for the cyclotide. For the disulfide-cyclized version and the linear version, activity also dropped to different degrees, 2 to 18-fold, and 1 to 30-fold respectively. Despite the massive difference in stability, the linear peptide still showed superior antimicrobial activity. The cyclotide and the disulfide-cyclized version demonstrated a slower bactericidal effect than the linear version. All three peptides were stable at high and low pH, and had very low hemolytic and cytotoxic activity.

## 1. Introduction

Among the most serious problems health care is facing is the increasing number of infections caused by antibiotic-resistant bacteria that can no longer be treated with previously effective antibiotics. In 2013, the World Health Organization (WHO) identified the development of antibiotic resistance as one of the major global threats to human society. Especially problematic are the so-called ESKAPE pathogens, with the six letters of ESKAPE representing the genera of the following bacteria: *Enterococcus faecium*, *Staphylococcus aureus, Klebsiella pneumoniae, Acinetobacter baumannii, Pseudomonas aeruginosa,* and *Enterobacter* spp [1]. Alternatives for antibiotics, especially with novel modes of action, are urgently needed [2].

One promising class of new compounds is antimicrobial peptides (AMPs). AMPs have some much-desired features, including a low chance of developing drug resistance and having fast acting, broad-spectrum activity, including against multidrug-resistant bacteria. So far, only a few have been investigated in clinical studies [2,3]. There are several databases specific for antimicrobial peptides, for example APD3 [4], which contains more than 3000 natural and some synthetic peptides, DBAASP, with >14,500 monomeric peptides, where >12,000 are synthetic [5], and DRAMP, with 19,899 entries, whereof 14,739 are patent entries, which tend to be synthetic peptides [6], with the majority of peptides in all databases being cationic. Although AMPs have an enormous variety of sequences and structures [7], they share certain common features. Cationic antimicrobial peptides are typically between 5 and 50 amino acids in length, with at least one excess positive charge due to lysine and arginine residues, and contain hydrophobic amino acids. AMPs are often stabilized by disulfide bridges, especially in defensins, that are composed of six cysteines forming three disulfide bonds [8]. In peptide design, cysteine bridges play a role to stabilize the peptides in terms of proteolytic and thermal stability [8].

In the last two decades of AMP research, it has become clear that these molecules have multiple biological activities, including antibacterial, antifungal, antiviral, antiparasitic, anticancer and immunomodulatory [9]. During the same time period, multiple bacterial targets of AMPs were discovered [10], including binding to RNA, DNA, or histones [11,12,13,14], binding to ATP, blocking DNA-dependent enzymes [15,16], blocking the synthesis of important outer membrane proteins [17], as well as binding to the chaperone DnaK, the ribosome [18,19], or lipid 2 [20,21]. 

Compared to many drug classes with lower molecular weight, peptides typically have poor oral bioavailability. That is coupled with a need for higher concentrations in the blood stream to achieve antimicrobial activity compared to many other highly active drugs that act on targets, receptors for example, at a nanomolar concentration. In order to reach high concentration at the site of infection, intravenous, intramuscular, or topical applications have the highest chance of success. In addition, application with osmotic pumps have shown promise [22]. Therefore, studies have been performed to investigate the interaction of AMPs with blood [23].

**Figure 1:**
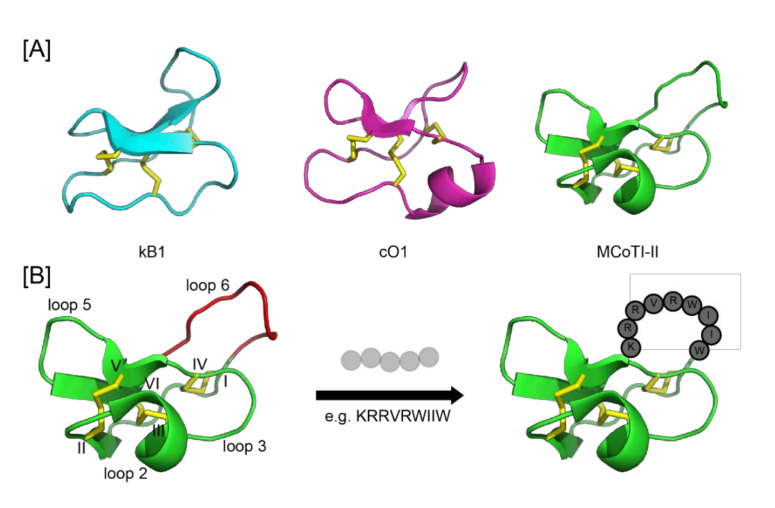
Structures of cyclotides. (**A**) Cartoon representations of the Moebius cyclotide kalata B1 (kB1, PDB ID: 1NB1), the bracelet cyclotide cycloviolacin O1 (cO1, PDB ID: 1NBJ), and the Momordica trypsin inhibitor II (MCoTI-II, PDB ID: 1HA9). (**B)** The principle of cyclotide grafting, showing the cyclic cystine knot motif with disulfide bonds highlighted in yellow. Cysteine residues are numbered in Roman numerals and intercysteine loops labelled. Loop 6 (highlighted in red) can be used to stabilize linear epitopes, such as the antimicrobial peptide optP7 used in this study. This concept, known as molecular grafting, results in engineered cyclotides with a range of desired bioactivities.

Cyclotides [24] are disulfide-rich macrocyclic peptides, so far found in plants from the Rubiaceae, Violaceae, Fabaceae, Solanaceae, and Cucurbitaceae plant families, with a single plant containing more than 100 different cyclotide homologs [25]. Their natural function is as host defense agents based on their reported activity against *Helicoverpa* insects [26] and a range of other pests, including nematodes, mollusks, and fungi [27]. Their structures comprise a macrocyclic backbone of around 30 amino acids, including six conserved cysteine residues connected in a cystine knot motif, in which two disulfide bonds and their connecting backbone segments form a ring that is threaded by the third disulfide bond [24] (Figure 1). Between the cysteine residues, the backbone loops are hypervariable, leading to the idea that cyclotides can be regarded as a natural combinatorial framework, with different cyclotides presumably targeting different pests [28].

Because of their exceptional thermal, chemical, and proteolytic stability, cyclotides have been proposed as scaffolds for peptide engineering and drug design [29]. Specifically, the idea that a bioactive peptide epitope could be grafted into a cyclic scaffold, maintain its activity, but be stabilized, has been pursued in several studies over the last few years [29] (Figure 1B). In these studies, there are options for various subtypes of scaffolds. Cyclotides are divided into three subfamilies: the Möbius, bracelet, and trypsin inhibitor cyclotides (Figure 1A). So far efficient chemical synthesis of cyclotides has been achieved for the Möbius and trypsin inhibitor subfamilies, but access to synthetic bracelet analogues remains challenging. Cyclotide grafting has been extensively reviewed [29], and recent examples include applications to anti-cancer therapeutics, pain, obesity, and cardiovascular disease.

Several naturally occurring cyclotide sequences have been proposed to have antimicrobial activity [30]. However, the cyclotides tested in that study were synthetically made and had rather weak activity against a range of common pathogenic bacteria, including *Escherichia coli* and *Staphylococcus aureus*, but were only active in low salt media, losing all activity under physiological conditions [30]. A number of other studies have also reported antibacterial activity for some natural cyclotides or synthetic derivatives of them [31,32,33,34]. For example, fractions comprised of cyclotides cyI3–cyI6 exhibited MIC values of 18 and 35 μM against *E. coli* and *K. pneumoniae*, respectively [33], cyclotide cycloviolacin O2 showed an MIC of ∼9 and 2 µM against *S. enterica* serovar Typhimurium LT2 and *E. coli*, respectively, and >50 µM against *Staphylococcus aureus* [34]. One study showed that a variant of cycloviolacin O2, while being only mildly active in vitro against *Staphylococcus aureus*, was effective in an animal infection model following *S. aureus* infection [35]. Therefore, it appears that most natural cyclotides have no to moderate antimicrobial activity, except under specialized conditions. However, some of the published MIC values show promise and further investigations are needed.

While cyclotides have been applied in more than 30 grafting studies for diseases ranging from cancer [36] to multiple sclerosis [37], there have, to our best knowledge, so far been no attempts to graft an antimicrobial peptide onto a cyclic scaffold. The aim of the current study was to graft a 9mer potent antimicrobial peptide (KRRVRWIIW-CONH_2_) [38] into a stabilized cyclotide framework to investigate whether the antimicrobial activity could be maintained in in vitro antimicrobial assays. The 9mer AMP was grafted into loops 5 and 6 of MCoTI-II. These loops were previously found to be able to accommodate a range of bioactive sequences [29]. MCoTI-II, a trypsin inhibitor cyclotide, was chosen for the grafting because it does not exhibit any antimicrobial activity [39] and does not show hemolytic activity towards red blood cells [40]. A 9mer antimicrobial peptide that is currently being investigated and developed in the Hilpert group was chosen because of its high potency and low toxicity [38]. The grafted cyclotide is compared to the linear 9mer peptide optP7 and a simple disulfide-cyclized 9mer (with two beta-alanine spacers and a cysteine at each terminus) version, c-optP7.

Figure 1B shows MCoTI-II, illustrating its three-dimensional structure and cystine knot motif, along with a representation of the grafting process, whereby a peptide can be inserted into one of the six backbone loops. In the study described here, the grafting of the antimicrobial peptide optP7 was successfully done in loop 6.

## 2. Materials and Methods

### 2.1. Peptide Synthesis and Purification

**Figure 2:**
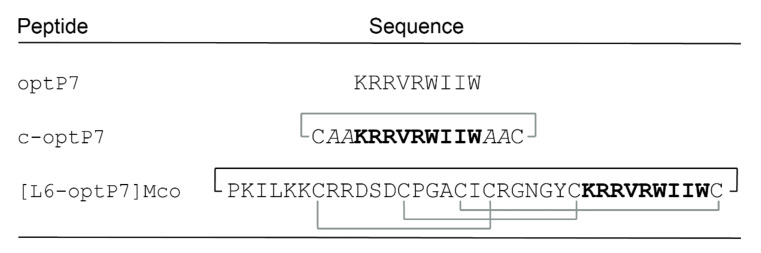
Peptides used in this study. The linear peptide optP7 was disulfide-cyclized (c-optP7), as well as grafted into loop 6 of the MCoTI-II scaffold ([L6-optP7] Mco). Disulfide-linkages are shown in grey, backbone cyclization of [L6-optP7]Mco is shown with a black line. Italic font denotes β-alanine residues.

MCoTI-II grafts were synthesized using Boc (tert-butyloxycarbonyl) based solid phase peptide synthesis (SPPS) on Boc–Gly–PAM resin (100–200 mesh; 0.5 mmol scale) on an automated peptide synthesizer (CS-Bio, Menlo Park, CA, USA). An S-trityl-β-mercaptopropionic acid thioester linker was used, and a standard hydrogen fluoride (HF) protocol (9 mL HF and 1 mL p-cresol for 1 h at 0 °C) was used for concomitant removal of side-chain protecting groups and cleavage of the peptide chain from the resin. Peptides were cyclized and oxidized overnight in 0.1 M NH_4_OAc with the addition of 2 mM oxidized glutathione at pH 8.5. Crude linear and cyclized oxidized peptides were purified by preparative reverse phase HPLC, applying a 1%/min gradient of solvent B (90% acetonitrile, 0.05% trifluoroacetic acid in water, *v/v*). Mass and purity of the peptides were confirmed by liquid chromatography electrospray ionization mass spectrometry and MALDI (Figure 2).

The short antimicrobial peptide optP7 was synthesized as described before by automated solid-phase peptide synthesis (SPPS) on a MultiPep RSI Peptide Synthesizer (INTAVIS, Tuebingen, Germany) using the 9-fluorenyl-methoxycarbonyl-tert-butyl (Fmoc/tBu) strategy [41]. Briefly, for automated SPPS, four equivalents of Fmoc amino acids (Bachem, Bubendorf, Switzerland) were coupled on TentaGel^®^ HL RAM resin (25-μmol scale, loading 0.3–0.4 mmol/g; Rapp Polymere, Tuebingen, Germany). Peptide amides were cleaved from the resin with 95% (*v/v*) aqueous trifluoroacetic acid solution (TFA, Fisher Scientific, Loughborough, United Kingdom) containing 5% (*v/v*) triisopropylsilane (TIPS, Thermo Fisher Acros Organics, Geel, Belgium)/water (1:1) scavenger mixture within 3 h. Crude peptides were purified to homogeneity of >92% by preparative RP HPLC on a Shimadzu LC2020 system equipped with a Jupiter 10 μm Proteo C18 column (90 Å, 250 × 21.2 mm, Phenomenex, Torrance, CA, USA) using a linear gradient system containing 0.01% (*v/v*) TFA in H2O (solvent A) and 0.01% (*v/v*) TFA in acetonitrile (solvent B). Pure products were finally characterized by analytical reverse phase high performance liquid chromatography (RP-HPLC) and liquid chromatography–mass spectrometry (LC–MS). The cyclized optP7 peptide was purchased at SynPeptide CO., LTD (Shanghai, China). 

### 2.2. NMR Sample Analysis

^1^H-NMR measurements of [L6-optP7]Mco (~1 mg in 500 µL) in 90% H_2_O, 10% D_2_O (*v/v*) at pH 3.5 were performed using a Bruker Avance-600 spectrometer at 298 K. Solvent suppression was achieved using excitation sculpting with gradients. Spectra were processed using TopSpin 3.6.1 software (Bruker, Billerica, MA, USA).

### 2.3. Bacterial Strains

The following strains were used in this study: *Escherichia coli*, ATCC 25922, *Staphylococcus aureus*, epidemic methicillin-resistant EMRSA-15, *Klebsiella pneumoniae*, clinical isolate from St George’s, University of London (SGUL, London, UK), *Acinetobacter baumannii*, clinical isolate from SGUL, *Pseudomonas aeruginosa*, PA01 wildtype strain, and *Enterococcus faecalis*, vancomycin-resistant ATCC 51299.

### 2.4. Antimicrobial Assays

The minimal inhibitory concentration (MIC) was determined in a microdilution assay using Mueller–Hinton (MH) or Brain Heart Infusion Broth (BHI) broth following a previously published protocol [42]. Briefly, a twofold serial dilution of the antibiotics and peptides were prepared and added to a bacteria solution, resulting in 2–5 10^5^ CFU/mL. The microtiter plates (polypropylene, Corning, Corning, NY, USA) were incubated for 18 h at 37 °C and MICs were taken visually. Based on our experience with proline rich peptides, their low activity in Mueller–Hinton broth, and their strong activity in diluted broth, we decided to also measure the activity of the peptide set in a 1/5 dilution of the broth. MICs in the presence of serum were established using human serum (Zen-Bio, Durham, NC, USA) and heat-inactivated serum (Sigma-Aldrich, St. Louis, MO, USA). A total of 20% (*v/v*) of each human serum was mixed with bacterial solution and added to predetermined concentrations of antibiotics and peptides, resulting in a final 10% (*v/v*) concentration of serum and 2–5 × 10^5^ bacteria.

### 2.5. Cytotoxicity Assays

HEK-293 (ATCC CRL-1573) cells were maintained in Dulbecco’s Modified Eagle Medium (DMEM), supplemented with 10% fetal bovine serum (FBS), 5% L-glutamine, and 5% penicillin–streptomycin (Merck Life Science UK Limited, Dorset, UK). The cytotoxicity assay was performed with the above media, but in the absence of penicillin–streptomycin. About 75,000 cells per well were seeded onto black, clear bottom 96-well microtiter plates (Greiner Bio-One, Kremsmünster, Austria) and left to incubate overnight at 37 °C, with 90% humidity and 5% CO_2_ conditions. The following day, media was removed and replaced with 100 µl fresh media and 100 µL of peptides at predetermined concentrations. A 0.2% Triton — X solution and sterile demineralized water served as positive and untreated controls. The plate was further incubated for 4 h. Then, 20 µL of 0.1 mg/mL solution of resazurin (Merck Life Science UK Limited, Dorset, UK) was added across the plate and left to incubate overnight for about 18 ± 2 h. The next day, fluorescence was measured at 535/595 nm excitation/emission using a Tecan INFINITE 200 PRO spectrophotometer (Tecan Group Limited, Männedorf, Switzerland).

### 2.6. Hemolytic Assay 

Fresh human blood was drawn into a citrate vacutainer (BD), and centrifuged at 1000× *g* for 5 min to isolate erythrocytes. The blood was rinsed a further three times with PBS and centrifuged at 1000× *g* each time, and was then resuspended in PBS to 4% (*v/v*). A total of 100 µL of erythrocyte suspension was added to a round bottom, polypropylene 96-well plate (Corning, Corning, NY, USA), containing predetermined concentrations of peptides. In addition, 0.1% (*v/v*) Triton — X 100 and PBS served as a positive (100% lysis) and negative (0% lysis), respectively. Plates were then incubated at 37 °C for one hour. Subsequently, the plate was centrifuged (1000× *g* for 5 min) and the supernatant was transferred to a clear, flat bottom microtiter plate (Scientific Laboratory Supplies Ltd, Nottingham, UK). The release of haemoglobin in the supernatant was measured at an absorbance of 450 nm using a Tecan INFINITE 200 PRO spectrophotometer (Tecan Group Limited, Männedorf, Switzerland).

### 2.7. Time Kill Assay

Overnight culture of *E. coli* (ATCC 25922) was diluted for approximately two hours to achieve logarithmic phase. The logarithmic phase inoculum was then adjusted to 1 × 10^6^ CFU/mL in 20% MH broth. Within a 1.5 mL microfuge tube (STARLAB (UK) Limited, Milton Keynes, UK), the peptides were added to the inoculum at a concentration that was threefold the MIC against *E coli*. The tubes were placed in a shaking incubator (225 rpm) at 37 °C. At predetermined time points between 0 and 24 h: 10, 20, 40, 60, 120, 240, and 1440 min for the peptides, and for the untreated control: 0, 60, 120, 240, and 1080 min, 100 µl samples were taken out. Furthermore, a 1 in 10 dilution series using sterile 10 mM Tris buffer (pH 7.5) was performed using the removed sample, then 5 × 5 µL were spotted for each dilution onto MH agar plates and incubated at 37 °C. The next day, the colonies were counted and the colony forming units, CFU/mL for each time point, were determined.

### 2.8. Stability Assays

Serum stability assays were performed in 100% human serum (Sigma-Aldrich, St. Louis MO, USA, human male AB plasma). Serum was centrifuged to remove lipids and incubated with the peptides at 100 μM for 0, 0.5, 1, 3, 6, and 24 h at 37 °C. Serum proteins were precipitated by adding 3 μL of TFA to 40 μL of peptide – serum sample (final TFA 7%). The remaining supernatant was diluted with 50:50 solvent A/B. The amount of peptide remaining (%) was quantified by UPLC-MS analysis. For pH stability experiments, peptides were incubated at a concentration of 20 μM at pH 1.2 and 7.5 for 24 h, and stability was monitored by UPLC analysis.

## 3. Results

### 3.1. Design and Synthesis of AMP Grafted MCoTI-II 

The target peptide optP7 was synthesized as a positive control, along with a disulfide-linked analogue where the peptide was cyclized via a disulfide-bond between C- and N-terminal cysteine residues, flanked by two β-alanine residues serving as spacers (c-optP7). The 9mer AMP was also grafted into loops 5 and 6 of MCoTI-II. These loops were previously found to be able to accommodate a range of bioactive sequences [29]. The crude peptides obtained from resin cleavage were analyzed by mass spectrometry, but only the loop 6 graft was found to be successfully synthesized and named [L6-optP7]Mco. Figure 2 shows the peptides that were used for the antimicrobial activity testing.

**Figure 3:**
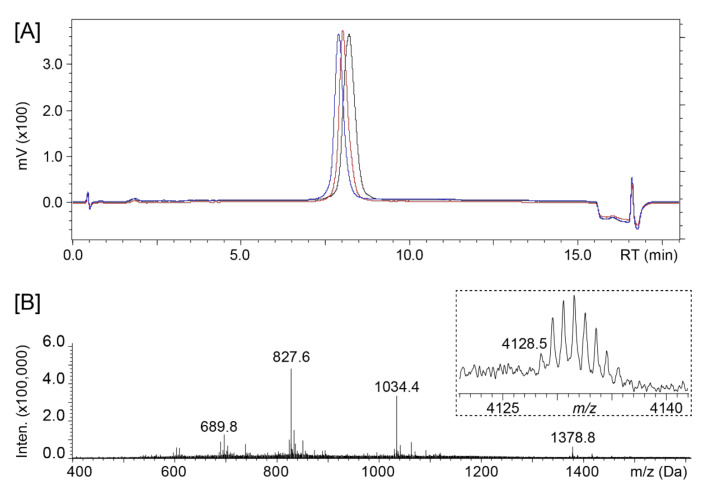
Characterization of [L6-optP7]Mco. (**A**) Analytical HPLC traces of the three pure fractions (f4–f6) and (**B**) ESI-MS spectrum of main fraction f6 showing [M + H]^+3^, [M + H]^+4^, [M + H]^+5^, and [M + H]^+6^ ions. MALDI insert shows the monoisotopic mass corresponding to the fully oxidized and backbone cyclized peptide.

A one-pot cyclization/oxidation resulted in a fully oxidized peptide that was purified via reverse phase HPLC. One-dimensional proton NMR spectra were recorded for three pure fractions f4 to f6 that showed the expected mass corresponding to a backbone-cyclic and fully oxidized peptide (Figure 3), but none were found to have a pattern indicative of a well-defined structure. A previous study generating kalata B1 grafted NS2B–NS3 protease inhibitors against dengue virus showed potent activity of non-native disulfide bonded species [43]. Therefore, it was of interest to proceed with antimicrobial testing of all three fractions.

### 3.2. Antimicrobial Activity of the Peptides

Fractions f4 to f6 of [L6-optP7]Mco were initially tested against *P. aeruginosa*, *E. coli*, and MRSA (Table 1) using a rich media, Mueller–Hinton broth (MH), with all fractions displaying about the same moderate activity (within errors of the method). Since it was reported that native cyclotides are more active in low salt conditions, we also tested antimicrobial activity against *P. aeruginosa* in 1/5 MH and found a strong improvement of activity. Calcium (Ca^2+^) and magnesium (Mg^2+^) often have the strongest effect on the antimicrobial activity of AMPs; their theoretical concentration in MH broth is 20–25 mg/L for calcium and 10–12.5 mg/L for magnesium [44]. By diluting this, the concentration will be only 1/5, and that could explain gain of activity. In addition, dilution of nutrition factors like nitrogen, vitamins, carbon, amino acids, and sulfur will also lead to changes of growth, changes of gene expression, and activation of different pathways, which can influence the bacterial cell wall and membrane composition, making the bacteria more susceptible. The activity in diluted media for fraction 4 was 4–8-fold improved, for fraction 5, 15.5-fold, and for fraction 6, 7.8-fold. This was encouraging since native MCoTI-II does not exhibit any antimicrobial effects [39] and, thus, further assays were carried out with sample [L6-optP7]Mco-f6, as more material was available for this fraction.

Based on the encouraging results from the first MIC testing, further testing was performed using a slightly modified set of ESKAPE pathogens (*Enterococcus* *faecalis*, *Staphylococcus aureus*, *Klebsiella pneumoniae*, *Acinetobacter baumannii*, *Pseudomonas aeruginosa*, and *Escherichia coli*) that are especially problematic for treatment because of the various multidrug-resistant strains. The results of the MICs are presented in Table 2. The ratios between c-optP7/optP7 and L-6-optP7-Mco/optP7, which translates to fold loss in activity compared to the linear peptide, are given in Table 3. Furthermore, MICs were determined in the presence of 10% human serum in order to study the effect of cyclization on stability in the presence of proteases in serum. The ratio of MH + 10% serum/MH and 1/5 MH + 10% serum/1/5 MH is given for comparison (Table 3). In addition, for *E. coli* and MRSA, we also tested if there is a difference between standard human serum and heat inactivated human serum. Heat inactivation effects mainly the complement system, part of the immune system that is able to enhance the action of antibodies and phagocytic cells and is able to kill bacteria. Because of limitations on the amount of material, some conditions which were highly unlikely to show activity were not measured.

Overall, the linear peptides showed strong antimicrobial activity against all ESKAP *(E. coli)* pathogens and the MIC values were very similar in broth versus 1/5 broth, except for *P. aeruginosa* (3.8-fold improvement) and MRSA (4-fold improvement). The activity of the simple disulfide cyclized peptide c-optP7 in broth was medium to weak. The activity improved strongly when diluting the broth; for *P. aeruginosa* this resulted in a 15.7-fold improvement, for *E. coli* an 8.9–17.8-fold improvement, and for MRSA a 15.7-fold improvement was observed. The peptide c-optP7 seemed slightly more active for Gram-negative bacteria, except for *K. pneumoniae*, with MICs of 9, 4–9, 71, and 4 versus Gram-positive bacteria with MICs of 18 and 18. The comparison between the linear peptides optP7 and the cyclized form c-optP7 shows decreased activity for all conditions, with a range of decrease between 5 and 88.8-fold. The average activity decrease factor was 25.5-fold. The grafted cyclotide [L6-optP7]Mco also showed medium to weak antimicrobial activity in broth. The activity improved in diluted broth: for *P. aeruginosa*, a 7.8-fold improvement; for *E. coli*, a 31-fold improvement; for *K. pneumoniae*, a 2-fold improvement; and for *A. baumannii*, a 15.5-fold improvement was observed. There also seemed to be slightly better antimicrobial activity against Gram-negative bacteria, except for *K. pneumoniae,* with MICs of 8, 2, 31, and 4 versus Gram-positive bacteria with MICs of 8 and 15. The comparison between the linear peptides optP7 and the hybrid peptide [L6-optP7]Mco showed decreased activity for all conditions, with a range of decrease between 1.3 and 41.3. The average activity decrease was 15.5-fold, 1.6-fold lower than for c-opt7, showing the potential of the cyclotide.

Changes of the MICs in the presence of serum showed surprising results, and the effect of the serum was influenced by the bacterium. For example, activity against *P. aeruginosa* in diluted broth showed that the antimicrobial activity in the presence of serum for the linear peptide was reduced 7.5-fold, for the cyclized peptide c-optP7 3.9-fold, and for the hybrid peptide 1-fold, indicating the improved proteolytic activity of the cyclotide analogue. However, the opposite was true for *E. coli*, where the linear peptide showed a loss of activity in serum by 1–2-fold, whereas the cyclic peptide lost 2.25–4.5-fold, and the cyclotide 7.5-fold. The addition of heat inactivated serum did not show changes in the MIC, except a two-fold reduction in MIC for c-optP7 against *E. coli*. The compliment systems seem not to be the reason for the decrease in activity of media vs. media and 10% serum.

**Figure 4:**
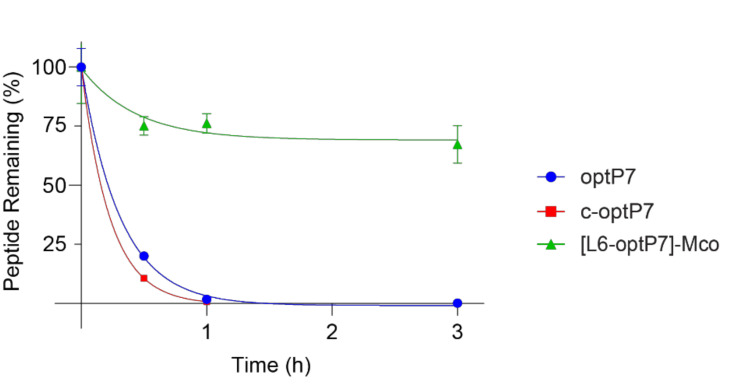
Stability of peptides in human serum. Percentage of peptide remaining over three hours, data shown as mean ± SD from experiments performed in triplicates.

We performed a serum stability assay to confirm if the cyclized and grafted cyclotide are more stable in serum and if the observed differences in activity in the presence of serum are correlated to the different stabilities of the peptides against serum proteases. While the linear optP7 and the disulfide-cyclized c-optP7 peptides are fully degraded after one hour, the cyclotide graft shows significantly improved stability (Figure 4), and about 30% of the peptide is still present after 24 h. The half-life of optP7 is 13 min, of c-optP7 9 min, and of [L6-optP7]-Mco 7 h and 15 min. This is in line with the previously reported stability of similar short antimicrobial peptides as well as cyclotides.

**Figure 5:**
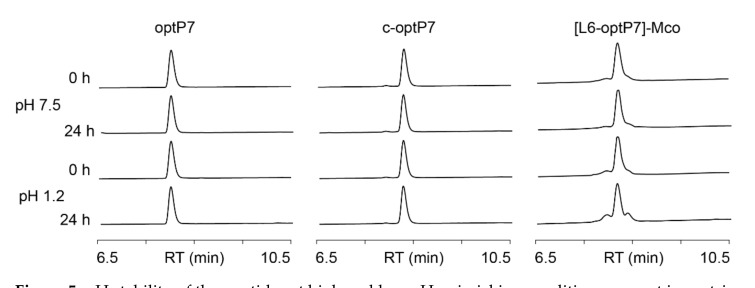
pH stability of the peptides at high and low pH, mimicking conditions present in gastric and intestinal fluids. RT = retention time.

To further analyze stability in regard to pH stability, the peptides were incubated at pH 7.5 as well as pH 1.2 to investigate their chance of survival in gastric and intestinal fluids. Notably, the linear and disulfide-cyclized peptides show exceptional stability over 24 h, and only the cyclotide graft shows minor signs of degradation after 24 h at low pH (Figure 5).

**Figure 6:**
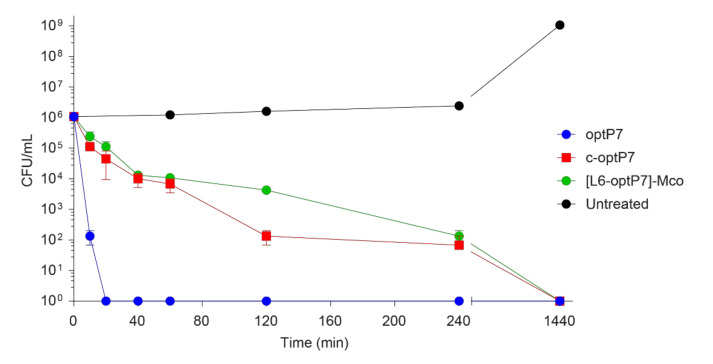
Time kill assay of three different peptides against *E. coli*, performed in 1/5 broth at 3 times the MIC. Curves represent the average of three experiments and the error bars show the standard deviation.

To investigate whether the cyclization and grafting of the linear peptide into a scaffold influenced the kinetics of killing, a time kill assay was performed three times against *E. coli* in diluted broth at three times the MIC. The results are presented in Figure 6.

The linear peptide optP7 is very fast acting and no survivor colonies were detected at 20 min and beyond. Interestingly, both the cyclized and the grafted version are drastically slower acting. This indicates that the structural rigidity and/or the absence of free termini changes their ability to kill the bacteria fast.

**Figure 7:**
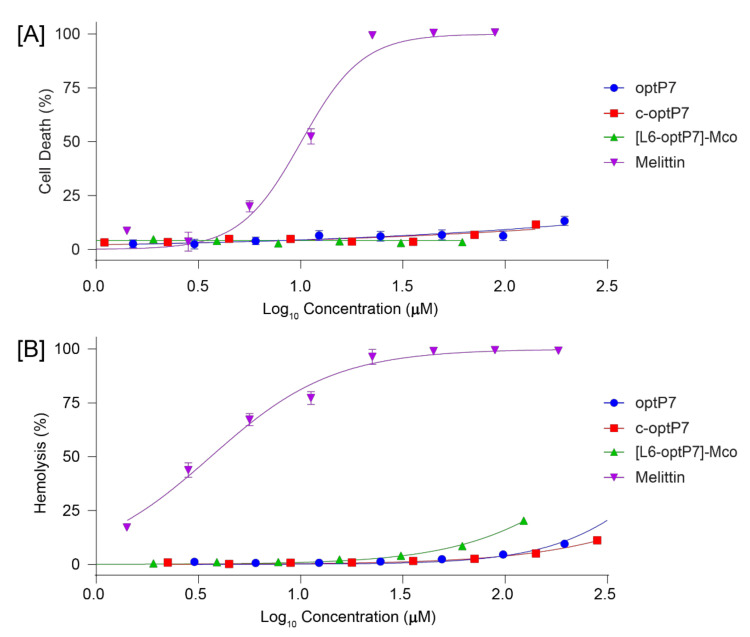
Cytotoxic (**A**) and hemolytic (**B**) activity measured against HEK-293 and human red blood cells in a dose-dependent manner.

Hemolytic activity and toxicity against HEK-293 for the three peptides were also determined, as seen in Figure 7. The peptide optP7 and the cyclotide MCoTI-II were chosen, in part, because of their low hemolytic and cytotoxic profile. Cyclization of optP7 and grafted optP7 did not increase toxicity and hemolytic activity. This data confirms the potential of all the candidates to be further developed.

## 4. Discussion

In comparison to the current COVID-19 crisis, antimicrobial resistance is currently a silent pandemic. The COVID-19 crisis shows very drastically the consequences of an infectious disease without an effective treatment option. Humankind could face an even more drastic pandemic when currently available antibiotics become ineffective, and no alternatives are found. Many operations, transplantations, and immunosuppressant therapies could not be performed if no effective antibiotics are available, leaving medical care devastated. Therefore, the development of new alternatives to treat bacterial infections is important and urgent.

Antimicrobial peptides are an interesting class of compounds to address new treatment options for multidrug-resistant bacteria [45]. Proteolytic stability and various toxicities of these peptides are challenging to overcome in terms of drug development. One strategy frequently used is cyclization, which can be established in various ways, for example, by introduction of cysteine bridges, head-to-tail cyclization, and other chemical modifications, such as lactam, hydrocarbon, or triazole bridges [46]. The introduction or natural occurrence of disulfide bonds can stabilize the active conformation of a peptide by reducing the energy cost required to bind target interfaces and increasing the affinity of the peptide to bind to and modulate them. Furthermore, the additional absence of free N- and C-termini protects such molecules from proteolytic degradation, as has previously been shown for native cyclotides [36] or engineered conotoxins [47].

We therefore followed two cyclization strategies: (i) a simple disulfide-cyclization by introduction of N- and C-terminal cysteines, and (ii) grafting onto an extremely stable scaffold with three disulfide bridges and backbone cyclization. The MCoTI-II cyclotide scaffold was used because of its inherent non-cytotoxic nature. In addition, MCoTI-II shows high stability in human serum [48,49]. A promising, in terms of strong broad-spectrum activity and very low cytotoxicity, linear peptide that is currently being developed was used to test the influence of cyclization. The linear peptide was introduced into loops 5 and 6, but synthesis could only be performed successfully when using loop 6. This result confirms the usefulness of our strategy to use multiple insertion sites to improve the chances of success. Fractions f4–f6 were investigated for their antibacterial activity against *P. aeruginosa*, *E. coli* and MRSA. All three fractions showed similar moderate activity. However, it has been reported that antimicrobial activity is salt dependent [30]. In addition, dilution of broth also leads to dilution in important nutrients and micronutrients that can change the metabolism and, consequently, composition of the cell wall and cell membrane, which could influence susceptibility of the bacteria towards the AMPs. Our own experience with proline–arginine rich peptides showed us that some AMPs have weak activity in broth, however, show good activity in diluted broth and, more importantly, in an infectious mouse model [50,51].

Indeed, the disulfide-cyclized as well as the cyclotide analogue showed, in all cases, an improvement in their antibacterial activity in diluted broth against the ESKAPE panel used here. Whether this activity is reflective of good efficacy in an infectious mouse model remains an open question. Pharmacological as well as toxicological parameters need to be determined first to judge the performance and potential of cyclized versus linear versions. Since this will take time and resources, a step backwards may be indicated first. Screening various linear peptides in the MCoTI-II scaffold with different spacers and developing a drug formulation might be the better approach. Once the activity is improved, animal models are more appropriate to use to judge the potential for further drug development. However, given the superior activity of the linear peptide, optimizing this peptide and its formulation seems to be the most attractive route to develop this AMP further, especially since the cost of goods are much lower and price currently still plays a great role in the antibiotic market.

The addition of 10% human serum resulted in a more complex result pattern than initially expected. The addition of serum brings in various ions, proteins, proteases, and complement factors, which can influence the action of a peptide and could initiate changes in bacterial gene expression, including a stress response. Because of the proteolytic stability of the MCoTI-II scaffold, for the grafted peptide, a low decrease in activity compared to no serum was expected. The loss of activity varied between the different ESKAPE organisms, from 1 to >8 fold. For the simple cyclized version and the linear version, activity also dropped to varying degrees, 2 to 18-fold and 1 to 30-fold, respectively. Most likely, the stress response of the bacteria, their protease production, changes in cell membrane and cell wall, as well as the presence of ions, proteins like albumin, and proteases are influencing the activity of the peptides. How relevant this is in an animal model and injecting the peptide directly in the blood stream needs to be evaluated, and that can be influenced by an optimized drug formulation.

A time kill experiment revealed that both cyclized variants are not able to kill the bacteria as fast as the linear peptide. The mode of action of the linear peptide has not been determined yet, but based on similar 9mer peptides, our working theory is that the peptide is able to depolarize and cross the membrane without destroying it. Once inside the cell, it can act through nonspecific binding to negatively charged proteins, ATP, DNA, or RNA that leads to cell death. Electron microscopy of similar 9mer peptides showed changes in the cytoplasm and the nucleoid, and showed that the cell membrane is still intact [41,52]. The free termini of the linear peptides seem to facilitate a better and faster membrane interaction and crossing compared to the cyclized variants.

Conclusions: In a proof-of-principle study, we demonstrated that it is possible to graft a short antimicrobial peptide into a cyclotide and retain some antimicrobial activity. The loop where the peptide is grafted into does matter. Simple cyclization and grafting a peptide into a complex cyclized molecule, like a cyclotide, reduces the antimicrobial activity compared to the linear variant, and also slowed the killing kinetics. By diluting the broth, antimicrobial activity was gained for the cyclic variants. The cyclotide showed a 33-fold increase in stability against degradation in 100% human serum compared to the linear variant. The addition of 10% human serum led to a decrease in activity for all three variants, albeit slightly less for the cyclotide. The linear variant outperformed both cyclic variants.

## Figures and Tables

**Table 1:** Minimum inhibitory concentrations in µM.

Bacteria	Growth Media	Fraction 4	Fraction 5	Fraction 6
*P. aeruginosa*	MH 1/5 MH	31 4–8	62 4	62 8
*E. coli*	MH	62	62	65
MRSA	MH	62	62	>62

Data are for *n* = 3 with modal values reported; MH represents Mueller–Hinton broth; MRSA = methicillin-resistant *Staphylococcus aureus.*

**Table 2:** Minimum inhibitory concentration (MIC) in µM.

Bacteria	Growth Media or *Ratio*	optP7	c-optP7	[L6-optP7]Mco	Melittin *
*P. aeruginosa* (Gram-negative)	MH	3	142	62	ND
	MH + 10% serum	49	>142	>62	ND
	*Ratio MH + 10%* serum*/ MH*	16.3	NA	NA	NA
	1/5 MH	0.8	9	8	ND
	1/5 MH + 10% serum	6	35	8	ND
	*Ratio 1/5 MH + 10%* serum*/ 1/5MH*	7.5	3.9	1	NA
*E. coli* (Gram-negative)	MH	0.8	71	62	16
	MH + 10% serum	6	>142	>62 *	ND
	*Ratio MH + 10%* serum*/ MH*	7.5	NA	NA	NA
	1/5 MH	0.4–0.8	4–9	2	3
	1/5 MH + 10% serum	0.8	18	15	11-22
	1/5 MH + 10% HI serum	0.8	35	15	11
	*Ratio 1/5 MH + 10%* serum*/ 1/5MH*	2–1	4.5–2	7.5	3.6–7.3
*K. pneumoniae* (Gram-negative)	MH	1.5	>142 *	62 *	ND
	MH + 10% serum	ND	ND	ND	ND
	*Ratio MH + 10%* serum*/ MH*	NA	NA	NA	NA
	1/5 MH	1.5	71	31	ND
	1/5 MH + 10% serum	24	142	>62	ND
	*Ratio 1/5 MH + 10%* serum*/ 1/5MH*	16	2	NA	NA
*A. baumannii* (Gram-negative)	MH	1.5	ND	62	ND
	MH + 10% serum	ND	ND	ND	ND
	*Ratio MH + 10%* serum*/ MH*	NA	NA	NA	NA
	1/5 MH	0.8	4	4	ND
	1/5 MH + 10% serum	3	71	31	ND
	*Ratio 1/5 MH + 10%* serum*/ 1/5MH*	3.75	17.8	7.75	NA
VRE (Gram-positive)	BHI	0.8	>142 *	>62 *	ND
	BHI + 10% serum	ND	ND	ND	ND
	*Ratio BHI + 10%* serum*/ BHI*	NA	NA	NA	NA
	1/5 BHI	0.8	18	15	ND
	1/5 BHI + 10% serum	24	>142	62	ND
	*Ratio 1/5 BHI + 10%* serum*/ 1/5BHI*	30	>7.8	4.1	NA
MRSA (Gram-positive)	MH	6	142	>62	4
	MH + 10% serum	49	>142	>62	ND
	*Ratio MH + 10%* serum*/ MH*	8.1	NA	NA	NA
	1/5 MH	1.5	18	8	0.7
	1/5 MH + 10% serum	6	>142	>62	1.4
	1/5 MH + 10% HI serum	6	>142	>62	1.4
	*Ratio 1/5 MH + 10%* serum*/ 1/5MH*	4	>7.9	>7.8	2

Data was obtained from experiments performed in triplicate, with modal values reported; asterisk represents a single measurement; serum = human serum; HI serum = heat inactivated human serum; ND = not determined; when calculation of ratio was not possible it was labelled as NA = not applicable; MRSA = methicillin-resistant *Staphylococcus aureus*; VRE = vancomycin-resistant *Enterococcus faecalis*; MH = Mueller – Hinton broth; BHI = brain heart infusion broth.

**Table 3:** Ratio of MICs of cyclized peptide versions over MICs of the linear peptide.

Bacteria	Growth Media	Ratio c-optP7/optP7	Ratio L6-optP7-Mco/optP7
*P. aeruginosa*	MH	47.3	20.6
	MH + 10% serum	NA	NA
	1/5 MH	11.3	10.0
	1/5 MH + 10% serum	5.8	1.3
*E. coli*	MH	88.8	77.5
	MH + 10% serum	NA	NA
	1/5 MH	10.0	3.3
	1/5 MH + 10% serum	22.5	18.8
*K. pneumoniae*	MH	NA	41.3
	MH + 10% serum	ND	ND
	1/5 MH	47.3	20.7
	1/5 MH + 10% serum	5.9	NA
*A. baumannii*	MH	ND	41.3
	MH + 10% serum	ND	ND
	1/5 MH	5	5
	1/5 MH + 10% serum	23.7	10.3
VRE	BHI	NA	NA
	BHI + 10% serum	ND	ND
	1/5 BHI	22.5	18.8
	1/5 BHI + 10% serum	NA	2.6
MRSA	MH	23.6	ND
	MH + 10% serum	NA	NA
	1/5 MH	18	8
	1/5 MH + 10% serum	NA	NA

For an MIC range, the average was determined and used for calculating the ratio. Serum = human serum; ND = not determined; when calculation of ratio was not possible, it was labelled as NA = not applicable; MRSA = methicillin-resistant *Staphylococcus aureus*; VRE = vancomycin-resistant *Enterococcus*; MH = Mueller–Hinton broth; BHI = brain heart infusion broth.

## Data Availability

All data are presented in the manuscript.
